# Phase Detection and Modulation Improvement for Active Load Modulation during Continuous Transmission

**DOI:** 10.3390/s21186155

**Published:** 2021-09-14

**Authors:** Nejc Suhadolnik, Jernej Rozman, Tilen Svete, Žiga Korošak, Maja Atanasijević-Kunc, Anton Pleteršek

**Affiliations:** 1 STMicroelectronics d. o. o., Tehnološki Park 21, 1000 Ljubljana, Slovenia; nejc.suhadolnik@st.com (N.S.); jernej.rozman@st.com (J.R.); tilen.svete@st.com (T.S.); ziga.korosak@st.com (Ž.K.); 2 Faculty of Electrical Engineering, University of Ljubljana, Tržaška Cesta 25, 1000 Ljubljana, Slovenia; maja.atanasijevic-kunc@fe.uni-lj.si

**Keywords:** wireless sensor networks, radio frequency identification, near field communication, active load modulation, binary phase shift keying, interference measurement

## Abstract

The paper covers one of the communication technologies used in wireless sensor networks. We have presented improvements in existing radio frequency identification (RFID) systems to address the problem of the phase selection in active load modulation (ALM). The phase selection affects the interoperability of communication devices and has to be addressed in the design phase of a new tag. A novel transmission method is presented to make the phase selection irrelevant for device interoperability. A second solution is shown to improve the existing system synchronization, which allows operation with arbitrary selected phase. A mathematical analysis of signals present on the antenna was used together with the reference reader model to perform an analysis of proposed improvements. We proved that the proposed transmission method is less affected by phase selection. Furthermore, we demonstrated that existing system improvement allows synchronization and operation at an arbitrarily selected phase despite the continuous transmission and large signal-to-interference ratio.

## 1. Introduction

Usage of wireless sensor networks (WSNs) has increased over the last decade, with multiple implementation options. WSNs differ in communication distance, network topology, device complexity and several other parameters. Each topology and technology used has their own advantages and disadvantages, which need to be taken into consideration when designing a WSN for a specific application. One of the studied communication technologies for WSNs is radio frequency identification (RFID) in the high-frequency (HF) band [1,2]. One of the main fields covering the HF RFID is NFC (near field communication), issued by the NFC Forum. Most of the NFC-related standards are intended for smartphones, and in 2019, two billion NFC-enabled devices were active [3]. The vast number of such devices can be an additional reason to implement NFC-based solutions in WSNs.

**Figure 1:**
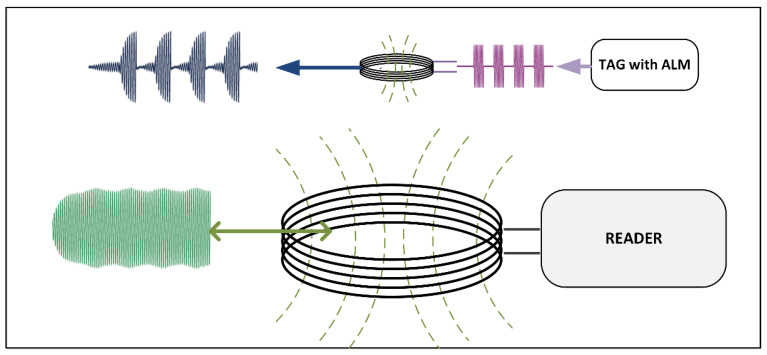
A communication pair composed of a reader and a tag with active load modulation (ALM). The tag transmits a modulated carrier signal, which is transformed into a magnetic field and combined with the reader’s magnetic field. When ALM is performed correctly, the reader cannot distinguish between a tag with ALM and a passive tag.

A communication pair of devices is composed of a reader and a tag in which the reader generates a magnetic field, which is modulated as a means of communication with the tag. The tag can use the reader’s magnetic field for its own supply and modulates the magnetic field to respond to the reader’s requests according to standard [4]. The important benefit of RFID sensor nodes is operation without a battery, which significantly reduces the complexity and cost of the sensor node. Furthermore, the sensor node can be highly integrated and composed only of a sensor, an integrated circuit and an inductive loop antenna. In certain cases, a battery is required for continuous sensor operation and data logging [5]. In these specific cases, battery power can be used to power the RFID interface and improve the communication range to the reader or reduce the tag antenna size as shown in [6,7]. The technique for the tag to actively transmit a magnetic field in such a way that the reader recognizes this magnetic field as modulation of its own magnetic field is called active load modulation (ALM). The communication pair is shown in Figure 1, where the reader generates its own constant magnetic field. A tag with ALM transmits a modulated carrier signal, shown with magenta color. The transmitted tag signal is converted to the magnetic field on the tag’s antenna and combined with reader magnetic field, shown with blue color. On the reader side, the tag’s signal is received and is seen as similar to passive load modulation (PLM). When the ALM is performed correctly, the reader cannot distinguish ALM form PLM. Due to this property, the term card emulation is sometimes used to describe ALM.

To fulfill the requirement that the reader recognizes the tag’s magnetic field as modulation of its own magnetic field, the tag needs to guarantee the phase and frequency alignment between both magnetic fields. The core of the ALM system is a circuit for phase and frequency synchronization. In its use today, the circuit is a phase-locked loop (PLL) where the loop is locked and is tracking the reader’s magnetic field phase when the tag is not transmitting. During transmission, the PLL phase tracking is stopped, and oscillator control is placed on hold. Depending on the oscillator stability, the loop needs to relock before the PLL phase drifts too far away from the reader’s magnetic field. The synchronization performances and system architecture are determined by the stability of the PLL frequency and PLL phase in hold mode, and the phase noise of the oscillator. The goal is to minimize the phase drift between the reader’s magnetic field and the tag’s transmitted magnetic field. For this purpose, a method has been proposed to measure the phase drift of a tag using ALM [8].

Recently, the majority of research in HF RFID and NFC has been focused on the power transfer, antenna modeling and use cases of the technology [9,10]. In the past, the scientific community has neglected the ALM topic and the majority, of research has been conducted within the industry, while the results of the research have been poorly reported. In recent years, a novel research on power amplifier efficiency and wireless transfer was reported using the same term, active load modulation [11,12,13]. This power amplifier and wireless power transfer work is not related to the topic of ALM in HF RFID and NFC.

Authors in work [14] presented a method and a circuit, which uses transmission gaps in the data frame to perform synchronization. This method works with lower PLL open-loop stability and has no limitation in the duration of the transmission frame, but it is limited by the antenna quality factor (Q). The proposed improvement of the active antenna damping in work [15] increases the useful range of the acceptable antenna Q factor. The drawback of the proposed method is the manual optimization of damping parameters for each antenna design and additional antenna interface to perform the damping. A different approach has been proposed in [16,17], where frequency and phase synchronization are achieved before the transmission frame is started. The drawback of this synchronization is the demand for a high PLL open loop stability, which can only be achieved with the use of an accurate external reference clock, such as a crystal oscillator. In certain use cases, the external clock is not available, and this limits the usefulness of such systems. The benefit of synchronization before transmission is the possibility of transmission without the synchronization pauses, thus increasing the effective transmission power.

A circuit presented in [18] uses an external clock for the reference and needs transmission gaps within the data frame for synchronization. This is a combination of two earlier presented systems and combines their drawbacks with no advantages. The presented system requires an external clock and an antenna with a low Q factor, which reduces the achievable communication range. 

**Figure 2:**
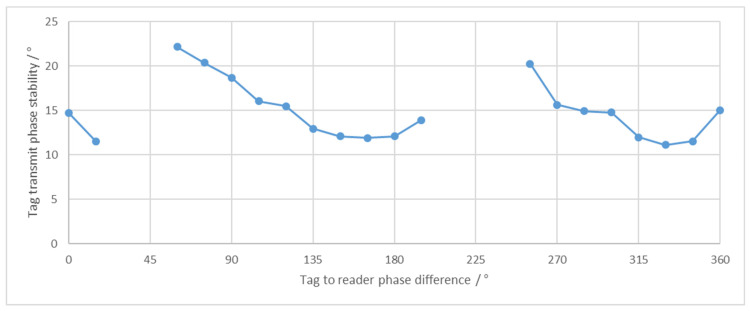
Phase stability of a system operating according to the principles presented in [19] was measured over a full range of tag to reader phase settings. At the set phases where no measurement results are shown, the system was not stable.

A different approach is proposed in [19], where transmission is continuous, without pauses, and no external clock is needed. The principle of operation is a measurement of the interference on the antenna between the received reader signal and the transmitted tag signal. The limitation of the proposed system is the narrow range of valid phase differences between the reader and the tag signal, which can be used for synchronization. We have performed a measurement of a system operating according to the principles presented in [19] for various reader to tag phase settings. The ratio between the received reader and transmitted tag signal was −25 dB. Results of tag phase error compared to ideal phase are shown in Figure 2. At phase settings where no results are shown, the system was unstable, and communication would fail. The main limitation for instability is the operation of the phase detector, which does not work over a complete input phase range. 

This paper addresses the issue of phase selection of the system presented in [19]. First, a novel transmission method is presented, which uses a combination of different carrier phases to improve the reception on the reader side, despite the limited phase difference selection. Second, an improvement of phase measurement is shown, which allows the selection of any phase difference between the reader and tag signal. 

This paper is organized as follows. In Section 2, basic concept of ALM is presented, a novel transmission method is described, and improvement of interference measurement is given. In Section 3, simulation results are shown for novel transmission and improved phase measurement. Discussion of the results is presented in Section 4.

## 2. Materials and Methods

A tag performs active load modulation by transmission of the magnetic field, which induces voltage on the reader antenna. Induced voltage from ALM is added to existing driving voltage present in the reader antenna. To achieve only amplitude modulation (AM) of voltage on the reader antenna, the appropriate phase difference between the reader driving voltage and the tag driving voltage needs to be set. The exact phase difference to achieve AM-only modulation between the two signals depends on the sum of multiple phase shifts in the system, such as phase shift in the power amplifier, reader antenna tuning and EMC filter (electromagnetic compatibility). Antenna detuning occurs when a resonant circuit, such as a passive tag, is placed in close proximity of the antenna or when a conductive material is placed in close proximity of the antenna. Inside a conductive material eddy, currents occur, which induce their own magnetic field and reduce the inductance of the coil composing the antenna. To accommodate for the phase shift, a fine-tuning of the phase shift is performed in the design phase of a tag to guarantee interoperability with targeted readers. The exact phase shift depends on the objective of the design. Usually, the target to achieve good interoperability is to have ALM signal on the reader side seen as part AM and part phase modulation (PM). This may not be sufficient, as the environment changes dynamically and introduces an additional phase shift by detuning of the reader antenna. Therefore, the signal can become AM only or PM only. This can present a problem for certain readers and can influence the communication quality. Several readers have, for historical reasons, implemented only AM receive channels or performed selection of only one channel for the duration of transmission frame. [20] The first version of NFC-analog specification [21] measured reader reception only with a reference listener, which was tuned to a carrier frequency and had a resistive load. This is known to generate AM-only modulation [22]. According to the first NFC forum release, the tag load modulation was observed only as AM modulation on a reference reader. In the latest NFC forum specifications [23], the reader is tested with a reference tag tuned to carrier frequency and to 16 MHz, while tag load modulation is measured as subcarrier spectral amplitude. Similar developments are present in EMVCo specification [24]. The importance of device interoperability has been observed by authors in [25,26], and interoperability always relays on testing against a collection of existing devices. Therefore, to achieve good interoperability operation against current standards is not sufficient. In cases when the signal is not present on both I and Q channels, the reader can choose the wrong channel, and communication fails. One solution is to readjust the phase difference after a failed communication. The described principles for reliable communication and good interoperability of ALM systems require a selectable phase shift on the tag side and a capability of the ALM system to synchronize at any phase difference. Another way to overcome the requirement of an adjustable phase difference is to transmit a signal that is always present at both AM and PM on the reader side regardless of the phase difference. To fulfill the latter, a novel modulation technique is proposed.

### 2.1. Orthogonal Phase Transition Shaping

**Figure 3:**
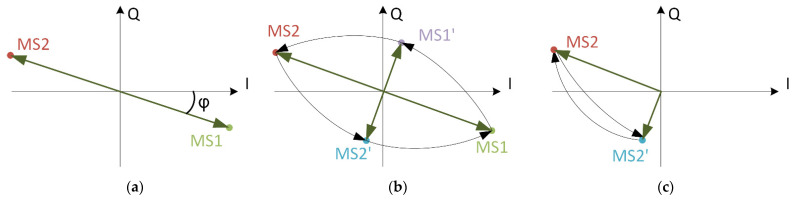
Subcarrier on the reader side is represented as modulated states (MS) for different transmissions. (**a**) Transmission, where the carrier phase is shifted for 180°—XOR mode. (**b**) Transmission with the orthogonal phase transition shaping (OPTS). (**c**) Special case of OPTS where MS2 is repeated as is the case of BPSK subcarrier modulation. MS1 and MS1’ are not shown in the diagram.

Subcarrier modulation of a carrier is shown on the I-Q diagram given in Figure 3a. The I-Q diagram is given from the reader’s perspective, where the phase difference between the reader signal and the received tag signal is an arbitrary value φ. The tag transmits two modulated states MS1 and MS2, which represent two subcarrier states and have the same amplitude and phase difference of 180°. There exists the phase difference φ, where both MS1 and MS2 will be aligned to I or to Q axis, resulting in modulation present only in I or only on Q channel. We can further try to find a combination of MS1 and MS2 with different phase and amplitude used for modulation, but there will always exist a phase difference φ where modulation is present only on I or Q channel on the reader side. 

The solution to avoid having only I or only Q channel modulation on the reader side is to use two intermediate modulated states MS1’ and MS2’, which are orthogonal to existing states MS1 and MS2. The I-Q diagram with additional modulation states is shown in Figure 3b. The tag transmits a short state MS1′ for each transition from MS1 to MS2 and transmits a short state of MS2′ for each transition from MS2 to MS1. The transition from MS1 to MS2 and back to MS1 with intermediate states can now be seen as an ellipse, which is always projected to both I and Q axes. This results in modulation on both I and Q channel, regardless of phase shift φ. The duration of intermediate state MS1’ and MS2’ has to be optimized for the particular carrier and subcarrier frequency. We call this modulation technique orthogonal phase transition shaping (OPTS).

Certain protocols define binary phase shift-keying (BPSK) modulation of a subcarrier. In these cases, MS1 or MS2 may be followed by the same modulation state, resulting in modulation, which is only present on I or Q channel during the repeated modulated state. To solve this problem, transmission of an intermediate modulation state is added between two repeated modulated states. This is visualized in Figure 3c, where two consecutive MS2 states are separated by MS2’. This technique guarantees that information is present on both reader channels regardless of the phase difference φ. It solves the problem of interoperability by reducing the phase difference dependence on the AM and PM reader received signal strength. To maximize the performance and to increase the signal strength on only one channel, a fine phase setting is still beneficial. The phase measurement improvement, which allows synchronization with any phase difference, is presented in the next subsection.

**Figure 4:**
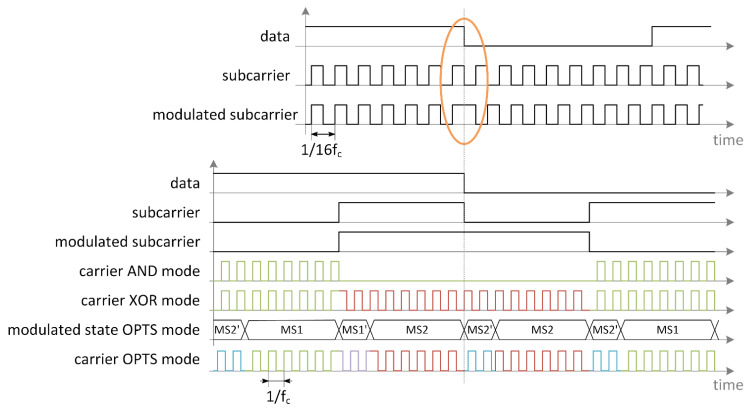
Timing diagrams for subcarrier and carrier modulation. In the upper part data, subcarrier and modulated subcarrier according to NFC B protocol are shown. In the lower part zoom of the section marked with orange is shown. Carrier modulation is shown for AND, XOR and OPTS mode. The carrier is colored according to modulation phase. Green carrier has reference phase, purple is 90° shifted, red is 180° shifted and blue is 270° shifted according to reference carrier.

Modulation in time domain is given in Figure 4. Data and subcarrier signals are shown in the upper trace. Modulated subcarrier is generated according to NFC B protocol. The section marked with orange ellipse is zoomed in on the lower trace. Modulated carrier is shown for three different transmission modes. The most basic transmission mode is AND mode, where the carrier is turned on and off. The second case is XOR mode, where the carrier signal is BPSK modulated, and carrier phase is inverted when modulated subcarrier is in a high logic state. This is the type of operation presented with phasors in Figure 3a. The last case is OPTS. The color of carrier signal corresponds to transmission phase, where green is the reference phase, purple is shifted for 90°, red for 180° and purple for 270°. For a modulated subcarrier transition, carrier is phase modulated according to I-Q diagram shown in Figure 3b. In the case, when data transition occurs, the modulated subcarrier value does not change. To guarantee signal on I and Q channel, the carrier is phase modulated according to diagram shown on Figure 3c.

### 2.2. Tag and Reader Antenna Model

**Figure 5:**
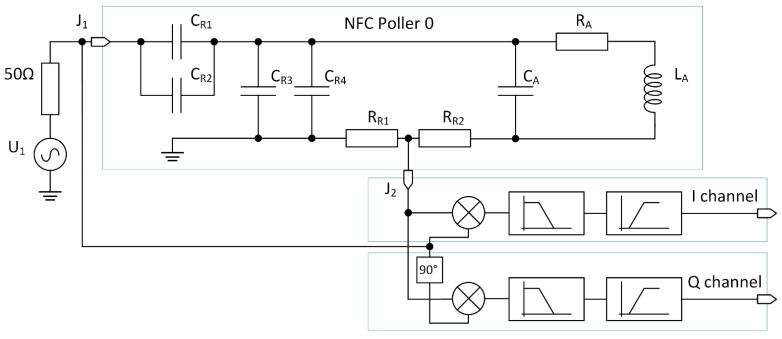
Reader model composed of NFC Poller 0, voltage stimulus, and I and Q channel model.

For the reader antenna model, we have used the NFC Forum specified Poller 0 antenna. The reader antenna schematic and model are shown in Figure 5. The standard specifies the antenna as the mechanical dimensions of the printed circuit board (PCB) traces. We have used Agilent E5071C network analyzer to measure and model the antenna coil (L_A_, C_A_, R_A_) for use in simulation. The capacitors C2 and C4 are specified as variable capacitors used to tune the antenna. A test method is described in the NFC specification to use a network analyzer and s-parameter measurement to trim the variable capacitor. Cadence SpectreRF sp analysis, which calculates the s parameters, was used. Values of obtained circuit elements are shown in Table 1.

To model a realistic reader I and Q channels, we used two mixers with 90° shifted clocks and analog filters. The filters are a series connection of a second-order low-pass filter with cut-off frequency at 3 MHz and a first-order high-pass filter with cut-off frequency at 50 kHz. Together, the filters form a band-pass filter and are needed to remove unwanted products of the mixing.

**Figure 6:**
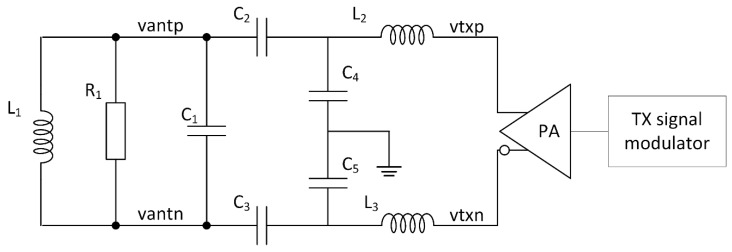
Tag antenna model, where L1, R1, C1, C2 and C3 form a resonant LC tank and L2, L3, C2 and C4 form a low-pass EMC filter. TX signal modulator and the power amplifier (PA) are driving the antenna.

The tag antenna model is shown in Figure 6. The D-class power amplifier output is connected to L2 and L3, which together with C4 and C5, form a low pass filter, used to suppress EMC emission. L1 represents the antenna coil, while R1 defines the antenna Q factor. Capacitors C1, C2 and C3 form the resonant capacitance and serve as a matching network between the low pass filter and the antenna LC tank. A coupling factor of 0.05 between the reader coil LA and the tag coil L1 was used for simulation purposes. The exact value of the coupling factor varies with distance and orientation between the reader and tag antennas. Therefore, any reasonable low coupling factor can be used to model the tag antenna for TX modulation purposes.

### 2.3. Phase Measurement Improvement

The second solution to the described problem of phase selection is to improve the system presented in [19] to allow synchronization at a selectable phase difference. On the tag side, a phase detector is needed to extract the reader to tag phase. The phase detector is capable of operation during continuous transmission and uses the interference in the tag’s antenna to its advantage. In the existing solution, the input phase range of the phase detector limits the selection range of tag transmission phase. The phase detector measures the phase difference between the transmitted tag signal and the tag antenna signal. The tag antenna signal is a superposition of the received reader signal and the transmitted tag signal, transformed by the tag’s antenna transfer function. The received reader signal has a noticeably lower value than the transmitted tag signal. The ratio between the received reader signal to the transmitted tag signal can be as low as −40 dB. Additionally, the transmitted tag signal is modulated with the subcarrier and data. 

**Figure 7:**
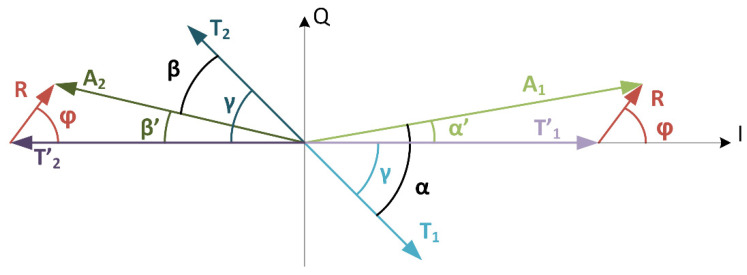
Phasors representing the voltages on the tag antenna during ALM transmission.

The settled voltage on the antenna at the end of the subcarrier half-period can be presented by phasors as shown in Figure 7. The right side of the I-Q plane shows tag signals at the end of the subcarrier half period. The transmitted tag signal is marked with T_1_ and represents the differential voltage at nodes *vtxp* and *vtxn* of circuit in Figure 5. The transmitted tag signal is transformed by the antenna transfer function H_ANT_, and we can write a transmitted signal on the antenna as T’_1_ = T_1_ |H_ANT_|e^j*γ*^, where *γ* is the phase shift of the antenna transfer function. The reader signal R on the antenna is added to the transformed transmitted tag signal T’_1_ and together they form the antenna voltage A_1,_ which is the differential voltage at the nodes *vantp* and *vantn* of circuit in Figure 5.

The phase difference *γ* between T_1_ and T’_1_ is the argument of antenna transfer function H_ANT_ and is composed of the phase delay of antenna resonance, antenna matching circuit and EMC filter. The transmitted signal T’_1_ is larger than the received reader signal R, and by taking this into consideration, we can write the phase difference *α*’ between T’_1_ and A_1_:

tan(*α*’) = |R| sin(*φ*)/(|T’_1_| + |R| cos(*φ*)). (1)

Equation (1) can be further simplified by omitting the cosine function in the denominator and approximating tan(*α*’) with *α*’. While the phase *φ* is not constrained, the phase *α*’ has a small value regardless of phase *φ* if the reader amplitude |R| is smaller than the tag’s antenna voltage |T’_1_|.

*α*’ ≈ |R|/|T’_1_| sin(*φ*); |T’_1_| >> |R|. (2)

Not all phasors are observable during transmission. We can observe the antenna voltage A and output of the power amplifier, the transmit signal T. The phase difference between the two phasors, which can be measured with a phase detector circuit, results in the equation:

*α* = *γ* + *α*′ ≈ *γ* + |R|/|T’_1_| sin(*φ*) (3)

In the next modulated state MS2, the transmitted signal T_2_ has opposite phase, and we can write an equation for angle *β*’ using the same approximation as for (2):

*β*’ = |R| sin(*φ*)/(|T’_2_| − |R| cos(*φ*)) ≈ |R|/|T’_2_| sin(*φ*); |T’_2_| >> |R|. (4)

The measurable phase *β* is:

*β* = *γ* − *β*′ ≈ *γ* − |R|/|T’_2_| sin(*φ*) (5)

Measurement of single phase *α* or *β* is obscured by the antenna phase shift *γ*. The movement of the tag or self-heating can change the electrical characteristic of the tag antenna and can introduce a change in the phase shift *γ*. The time difference between the measurement of phase *α* and *β* is one-half period of the subcarrier and is considerably shorter compared to the dynamics of self-heating or the tag movement. Therefore, the change of antenna transfer function H_ANT_ between two consecutive measurements is negligible. The transmitted signals amplitudes |T1| and |T2| can also be considered constant and the resulting transmit power can also be considered constant during this short time difference. We can replace |T’_1_| and |T’_2_| with |T’|, which is the transmitted signal amplitude increased by gain |H_ANT_|. By calculating the phase difference *α* − *β* we remove phase shift *γ* and double the sensitivity:

*α* − *β* ≈ 2 |R|/|T’| sin(*φ*) (6)

The amplitude of antenna signal at the end of modulated state MS1 and MS2 can be measured using a similar approach:

|A_1_|^2^ = [|T’_1_| + |R| cos(*φ*)]^2^ + |R|^2^ sin^2^(*φ*) (7)

|A_2_|^2^ = [|T’_2_| − |R| cos(φ)]^2^ + |R|^2^ sin^2^(φ) (8)

As the transmitted signal T’ is considerably larger than the reader signal R, we can neglect the second term |R|^2^ sin^2^(φ), and by assuming that the first term is always positive, we can square root both sides of the equations. If we use the approximations and calculate the difference between (7) and (8) we obtain:

|A_1_| − |A_2_| ≈ 2|R| cos(φ) (9)

In comparison to the phase measurement, the multiplier of the cosine function is not the same as the multiplier before sine function in Equation (6). By averaging measurements of |A_1_| and |A_2_|, we can retrieve the amplitude of |T’| and use it for the division of Equation (9) to obtain the same multiplier as in Equation (6). The results of the above operation are two measurements; one is the sine function of the phase difference φ and other is the cosine function of the same phase difference. At this point, an arbitrary method can be used to determine the phase difference. In case of an analog solution, this can be an angle interpolator, while in the form of a digital solution, a CORDIC (coordinate rotation digital computer) or any other algorithm to retrieve the phase difference can be used. 

## 3. Simulation and Results

### 3.1. Transmission Analysis

**Figure 8:**
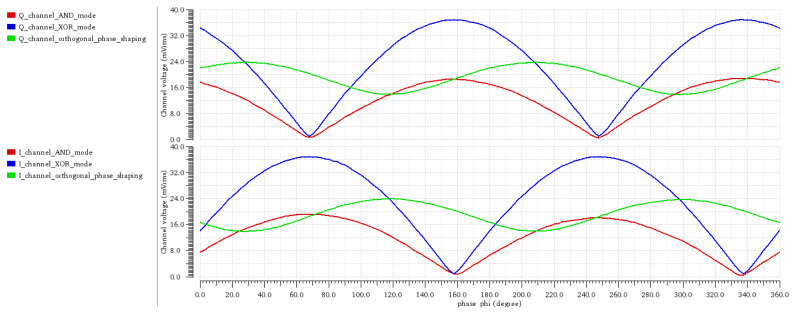
Reader I and Q channel rms voltage versus the tag transmission phase. The signal received by the reader is shown for three transmission modes: AND, XOR and the orthogonal phase shaping. The orthogonal phase shaping guarantees sufficiently large signal on the reader I and Q channels when compared to the other two modes.

We performed a computer simulation of the existing and proposed orthogonal phase transition shaping method with Cadence Spectre simulator. The NFC Forum type B protocol modulation was used for transmission with prescribed 13.56 MHz carrier frequency and 16 times smaller subcarrier frequency. The data was transmitted with 106 kbit/s, BPSK modulated subcarrier. To measure the effectiveness of different transmissions methods, the rms voltage was measured on I and Q channels of our reader model as a function of the tag transmission phase. Analysis for three different transmission methods were performed. The first was on-off keying of the carrier, according to the standard modulation scheme. This transmission mode was called AND mode and is shown in Figure 8 in red. This technique is closest to passive tag transmission and is used in systems presented in [7,14,15,18]. The second analysis was performed with the continuous carrier transmission, where the carrier phase was shifted for 180° according to the standard modulation scheme. The result is shown in Figure 8 with blue trace. This type of transmission is used in system [19] and it is called XOR mode. The technique used for the last analysis is presented in Section 2.1. The results of the simulation are given in Figure 8 in green color. The described transmission generation methods were implemented with a VerilogA model, represented by TX signal modulator on Figure 6. The duration of intermediate modulation states was optimized for the particular standard and was two carrier clock periods. Results for AND mode and XOR mode exhibit a gap where all signal information was present only on the I or Q channel, which can cause receiving problems on certain existing readers. The novel transmission with orthogonal phase shaping resulted in a signal, which is always present on both channels. The tradeoff compared to XOR transmission mode gave us a smaller maximum amplitude. This is beneficial as the signal variation versus the phase was significantly reduced.

**Figure 9:**
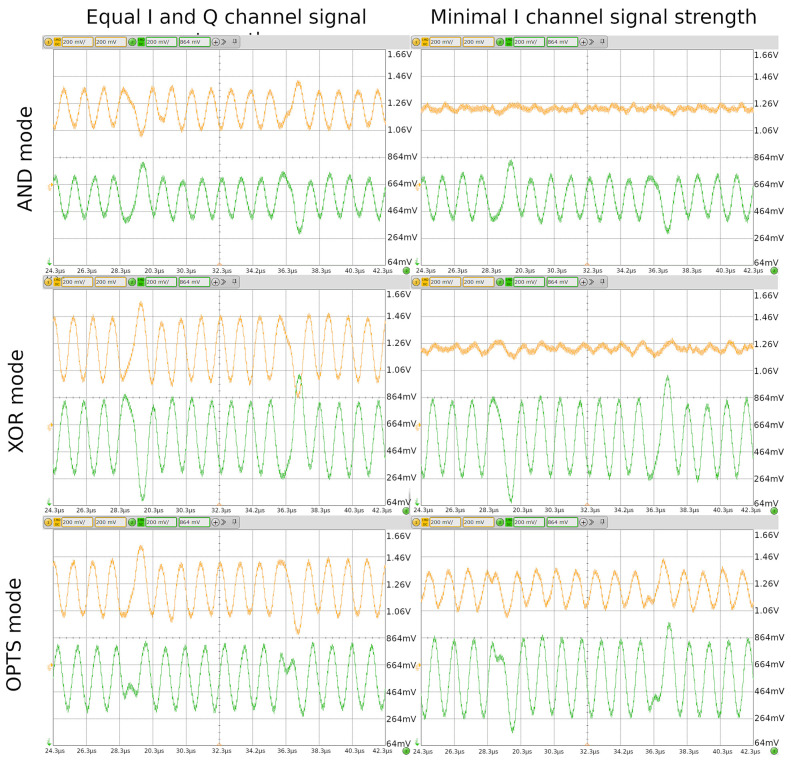
Measurements of reader I and Q channel for different transmission modes. I channel is shown with yellow trace and Q channel with green trace. Two extreme cases are displayed for each transmission mode. In the left column, transmit phase was selected to achieve equal I and Q channel; in the right column, transmits phase was set for minimum signal on the I channel. For the case of OPTS transmission, the signal was always present on I and Q channel, regardless of transmit phase.

To validate the proposed transmission method, we performed an experiment with a commercially available reader (ST25R3916), where we observed I and Q channel outputs. For transmission signal generation, we used Keysight 33500B arbitrary waveform generator and tag antenna, according to the circuit presented in Figure 6. For the tag antenna, we used a rectangular antenna with dimensions of 30 × 30 mm and 3 turns. We generated carrier modulations, presented in Figure 4, for AND, XOR and OPTS. Arbitrary waveform generator was loaded with generated carrier signals and was used to drive the tag antenna. The reader I and Q channel outputs were measured and are presented in Figure 9. For each transmission mode, two cases are shown. In the left column, the tag transmitted phase was set to achieve equal signal strength on I and Q channel. In the right column, the tag transmitted phase was set for a minimum signal strength on I channel. In the first row, the AND transmission mode was used, and clearly at a minimum, I channel phase setting signal was almost nonexistent. The same applied to the second row, where XOR transmission mode was used. The noticeable difference was increased amplitude on both channels for XOR mode. In the third row, OPTS transmission mode was used, and signal was always present on both I and Q channel. This was true for the case where the tag transmission phase was set to achieve minimum signal on I channel. At the subcarrier phase inversion, a glitch was observed, but was small compared to received signal amplitude. Compared to AND and XOR mode, OPTS had a lower overshot at subcarrier phase inversion.

### 3.2. Simulation of Improved Phase Detector

**Figure 10:**
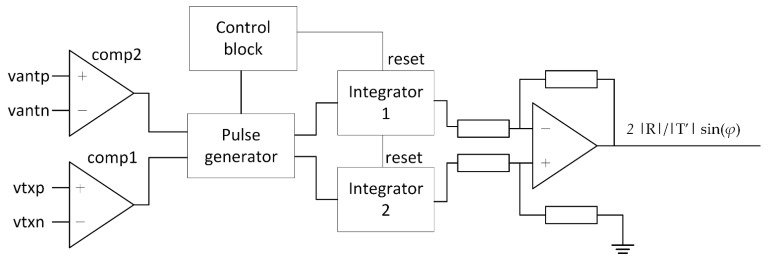
Circuit for phase difference measurement corresponding to Equation (6).

To perform the measurement of the phase between the reader and the tag based on the method presented in Section 2.2, a custom circuit was designed. The circuit was connected to the tag antenna model shown in Figure 6, which was coupled to the reader model shown in Figure 5. Due to the tag antenna topology, voltage on the antenna can reach several volts, which can be higher than the allowed voltage by the circuit technology node. In this case, an input divider was needed which, and for simplicity, is not shown in Figure 6. The circuit for phase measurement was composed of two comparators operating on the carrier signal frequency to perform clock extraction. The complete circuit is shown in Figure 10. The first comparator *comp1* was placed on the differential output of the power amplifier and extracted the transmission signal represented by phasors T_1_ and T_2_ in Figure 7. The second comparator *comp2* was connected to the coil of the tag antenna between nets *vantp* and *vantn*. The extracted clock is represented by phasors A_1_ and A_2_ in Figure 7. The phase difference between the extracted clocks was transformed to pulses with logic gates shown as the pulse generator, while the resulting pulses were converted from time to voltage with integrators. The reset and start of integration were controlled by a finite state machine shown as a control block. The control signals defined a time window of one carrier period at the end of a subcarrier half-period in which the integration was performed. Until the next measurement, the result of integration was kept on the integrator output. Two integrators were used, one to measure phase *α* and the second to measure phase *β*. The difference calculation of the two integrated voltages was implemented with an analog difference amplifier. The output of the difference amplifier corresponded to the result of Equation (6). 

**Figure 11:**
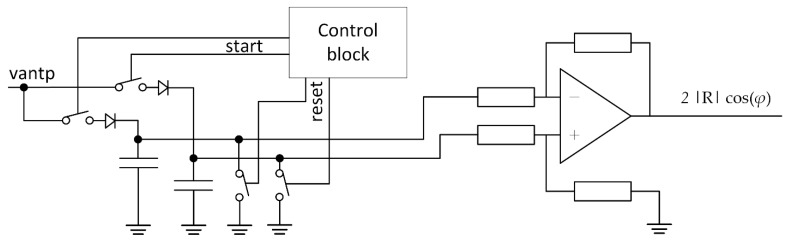
Circuit for amplitude difference measurement corresponding to the Equation (9).

Amplitude measurement was implemented with the sampling circuit shown in Figure 11. The circuit was composed of a switch controlled peak detector, which could be reset with a switch connected to the ground. A finite state machine, shown as a control block, controlled the start and reset of the peak detectors. Two peak detectors were connected to the difference amplifier, where the output of amplitude measurement corresponded to Equation (9). The gain of the difference amplifiers had to be set according to the specified operation range, or automatic gain control could be implemented to improve the operation range. The phase computation from amplitude and phase measurement is out of the scope of this paper and can be implemented either with analog or digital techniques. In the case of digital implementation, the adequate gain has to be set at the difference amplifiers such that the output can be directly sampled by an analog to digital converter. For the digital phase calculation, a CORDIC or any suitable algorithm should be used. 

**Figure 12:**
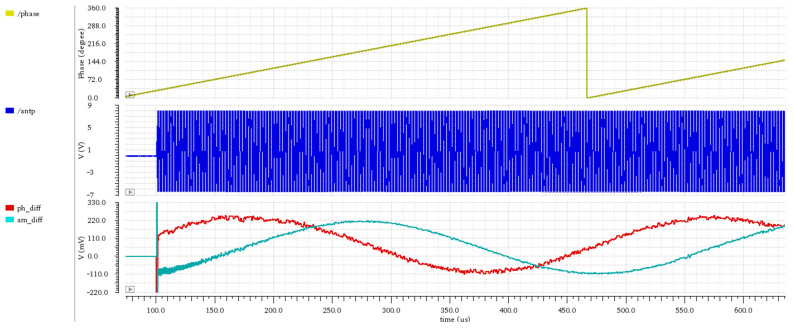
Simulation results for the phase and amplitude measurement system. In top subplot, the phase between the reader and the tag is shown, in middle subplot the voltage on the tag antenna is shown. In the bottom subplot, the phase measurement result (ph_diff) and amplitude measurement results (am_diff) are shown. Note the large difference between the antenna voltage before and after the transmission start at 100 us.

To verify the circuit performance, we designed the circuit in 90 nm CMOS technology (complementary metal-oxide-semiconductor). The simulations were performed with Cadence Spectre simulator. In simulation, we introduced a small frequency displacement between reader and tag clocks to obtain a linear phase drift. The analysis outputs are the phase and amplitude measurements shown in Figure 12. The results show clear cosine and sine functions of the reader phase, despite the large transmission signal on the tag antenna. The ratio between the received reader and the transmitted tag signals |R|/|T| used in the shown analysis was −35 dB. The phase and amplitude were measured only at the end of the subcarrier half-periods. Subtraction between two measurements samples resulted in an output sampled with a frequency of the subcarrier. 

**Figure 13:**
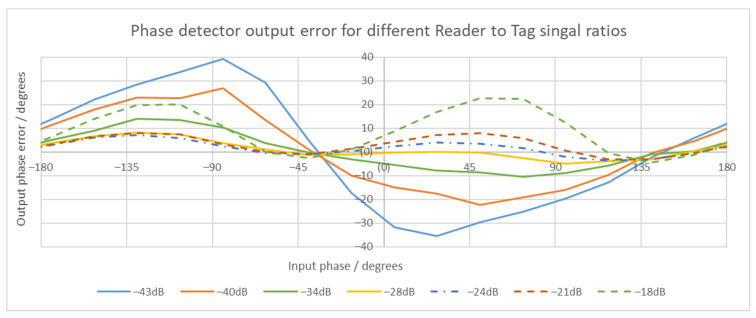
Phase detector output phase error over a full range of input phase. The lines represent different reader-to-tag signal ratios. Except for the −43 dB signal ratio, all phase errors have an absolute value lower than 30 degrees. The output of the phase detector is valid over complete input phase range.

The phase detector performance was verified for different reader-to-tag signal ratios with a second analysis, performed over a full input phase range. The results are shown in Figure 13. The input phase was the phase between the reader and the tag internal clock. The output phase error was calculated in post-processing using arctangent function of the amplitude and phase measurement. The phase detector output was valid for a complete input phase range. The absolute value of the output phase error was smaller than 30° for reader-to-tag signal ratios between −40 and −18 dB. For the ratio of −43 dB, the phase error could be up to 40°, which is higher than the 30° allowed in ISO 14443-2:2020 standard [27]. The output phase error was decreasing with increasing ratio and was minimal at ratio value of −28 dB. The output phase error increased for higher ratio values.

## 4. Discussion

The proposed OPTS transmission modulation has shown promising performance gain on reader receiver side across a full range phase shift compared to existing AND and XOR transmission modulations. It was shown that the method guarantees signal on the reader I and Q channel for all phase differences between the tag and the reader. The benefit of the OPTS modulation is improved interoperability and communication reliability. This advantage is important for the cases where the environment causes detuning of antennas, which results in the phase shift of tag signal on reader side, especially on the existing installed equipment that is not operating according to latest standards. The OPTS modulation can also be used on the tag with a clock synchronization system that does not have a circuit for fine phase adjustment or if it cannot operate at arbitrary phase shift. The presented transmission mode can simplify the design of a new active tag antenna, as the designers can omit the fine phase tuning design step. The drawback of this use case is a somewhat smaller ALM signal seen on the reader side. 

Our second presented solution, the improved phase detector, is capable of operation during continuous transmission. The dynamic range of the ratio between the received reader and the transmitted tag signal was shown to be between −40 and −18 dB. In this signal ratio, the range phase detector had limited phase error at the output over a full range of input phases. This phase detector operation can support a clock synchronization system to maintain a lock on reader signal regardless of phase shift between reader and tag. The ability to select the transmission phase enables the operation with XOR transmission modulation, where the tag transmission phase has to be carefully selected to guarantee the interoperability with existing equipment. The benefit of using XOR modulation is increased signal amplitude observed on reader side and increased communication range. The drawback is an additional design step in the development of a tag to guarantee the interoperability. By implementing both presented solutions, the end user designing a complete tag system with ALM has the option to decide between a simpler solution or a more complex one to increase communication range. For future work, we foresee further development of proposed techniques and implementation on an integrated circuit.

## 5. Conclusions

An overview of existing ALM solutions was given in the paper, focusing on signal synchronization between the received reader signal and the transmitted tag signal. The emphasis was placed on the limitation of the existing solution, which is able to synchronize during the continuous transmission without pauses for synchronization. The limitation of the existing system was identified as the phase detector, which has a limited input phase range. We have presented two solutions. One solution is the improved phase detector circuit for which we showed operation over a full input phase range. The phase detector has shown an acceptable phase error for the ratios between the received reader and the transmitted tag signals in the range from −40 to −18 dB. The second solution is a new transmission modulation with orthogonal phase transmission shaping (OPTS) to improve the signal reception on the reader side. We proved that the new modulation produces signal on I and Q channel inside the reader receiver, regardless of the signal phase between the reader and the tag. This is an improvement over existing solutions for which we showed that both exhibit a phase setting where the signal inside the reader receiver is present in only one, I or Q channel. This improvement is important for high interoperability with existing equipment.

## Figures and Tables

**Table 1:** Elements values used in models of the reader and the tag.

Reader Model Values			Tag Model Values		
Element	Value	Unit	Element	Value	Unit
C_R1_	2.62	pF	L_1_	850	nH
C_R2_	107	pF	R_1_	800	Ω
C_R3_	5	pF	C_1_	139	pF
C_R4_	62.8	pF	C_2_, C_3_	165	pF
R_R1_, R_R2_	2.7	Ω	C_4_, C_5_	560	pF
L_A_	670	nH	L_2_, L_3_	77	nH
C_A_	31.5	pF			
R_A_	1.35	Ω			
